# Evaluation of a novel ovarian cancer-specific fluorescent antibody probe for targeted near-infrared fluorescence imaging

**DOI:** 10.1186/s12957-020-01843-6

**Published:** 2020-04-06

**Authors:** Junchen Chen, Chen Zhang, Yanxiu Guo, Xiaohong Chang, Ruiqiong Ma, Xue Ye, Hongyan Cheng, Yi Li, Heng Cui

**Affiliations:** 1 Department of Obstetrics and Gynecology, Peking University People’s Hospital, No. 11, Xi-Zhi-Men South Street, Xi Cheng District, Beijing, 100044 China; 2 Center of Gynecologic Oncology, Peking University People’s Hospital, No. 11, Xi-Zhi-Men South Street, Xi Cheng District, Beijing, 100044 China

**Keywords:** Ovarian cancer, Near-infrared fluorescence imaging, COC183B2-800, Surgery

## Abstract

**Background:**

To meet clinical needs, fluorescence-guided surgery has emerged as a new technique that guides surgeons in the resection of cancerous tissue by highlighting tumour lesions during surgery. We aimed to evaluate the novel ovarian cancer-specific antibody fluorescent probe COC183B2-800 (COC183B2 conjugated with IRDye800CW) in tumour-specific imaging to determine if it can help surgeons remove malignant lesions under fluorescence guidance.

**Methods:**

The expression of OC183B2 antigen in epithelial ovarian cancer (EOC) tissues and cell lines was determined using immunohistochemistry (IHC). Western blotting was used to verify the expression of OC183B2 in SKOV3-Luc tumours. Antibodies against OC183B2 and mouse immunoglobulin G1 (IgG1) were conjugated with IRDye800CW to develop the antibody fluorescent probes COC183B2-800 and IgG-800 (immunoglobulin G1 conjugated with IRDye800CW). A subcutaneous mouse tumour model of SKOV3-Luc cells was constructed. Bioluminescent imaging (BLI) was conducted to detect the tumour location. Near-infrared fluorescence (NIRF) imaging was performed after the mice were injected with imaging agents. The mice were sacrificed 96 h postinjection, and the biodistribution assays were performed using NIRF imaging.

**Results:**

In 69 EOC patients, the total positive rate of OC183B2 in EOC tissues was 89.9% (62/69). Expression of the OC183B2 antigen was positive in SKOV3-Luc, 3AO, ES2 and A2780 cells. The OC183B2 antigen could be detected in SKOV3-Luc tumours. NIRF imaging of the COC183B2-800 probe at different doses showed a high fluorescent signal at the tumour location that was in line with the site detected by bioluminescent imaging. The tumour background ratio (TBR) was significantly higher in the COC183B2-800 group than in the IgG-800, IRDye800CW and PBS groups. The fluorescent probe COC183B2-800 is metabolized mainly through the liver and does not accumulate in other organs.

**Conclusions:**

COC183B2-800 shows effective tumour-specific targeting of EOC and is a promising diagnostic and therapeutic tool for fluorescence-guided surgery.

## Introduction

Ovarian cancer is the most lethal gynaecological malignancy. Most patients are diagnosed with advanced ovarian cancer, and early detection of ovarian cancer is difficult due to nonspecific symptoms or a lack of symptoms [1]. Surgery is one of the most frequent and effective treatments for cancer and is often the first and only curative option [2]. Intraoperative visualization of tumours, including metastatic and microscopic lesions, is of significant importance for the outcome of therapy [3].

Conventional imaging techniques include magnetic resonance imaging (MRI), computed tomography (CT), X-ray and ultrasound. However, the signal specificity and sensitivity of these imaging techniques are limited. Moreover, these imaging techniques cannot be conducted during surgery. Currently, surgeons mainly depend on visual inspection and palpation to identify lesions. The presence of occult lesions in the tumour margin and nonenlarged yet metastatic lymph nodes needs to be considered to determine how radical surgery should be. To detect these tiny lesions accurately during the operation, a highly sensitive intraoperative imaging modality is urgently needed. Near-infrared fluorescence (NIRF) imaging makes visualization of cancer a reality during the operation, and it could help realize complete resection of the tumour [2]. Furthermore, fluorescence imaging is safe and noninvasive with the advantages of high specificity and superior resolution.

We previously developed the monoclonal antibody COC183B2, which has high-affinity binding to epithelial ovarian cancer (EOC) tissue. It was produced using hybridoma technology in our laboratory. The antigen of COC183B2 is ovarian cancer-associated antigen OC183B2 [4, 5]. Our previous work showed that ^131^I-COC183B2 can localize EOC by radioimmunoimaging [6]. IRDye800CW is a NIRF dye that is cleared via the kidneys, and the extinction coefficient of IRDye800CW is high [7]. More importantly, IRDye800CW has already been extensively tested in clinical studies and proved safe in humans [8, 9]. We conjugated COC183B2 with IRDye800CW and synthesized COC183B2-800. The imaging agent of COC183B2-800 needs to bind specifically to antigen OC183B2 in the tumour, facilitating specific imaging.

In our study, we developed and validated a novel EOC antigen-targeting fluorescent probe, COC183B2-800, for tumour-specific imaging of EOC that has high clinical translational potential.

## Materials and methods

### Immunohistochemistry (IHC) staining of EOC tissues

IHC staining was conducted in the human EOC tissues of 69 patients using COC183B2 antibody as previously described [10]. Mouse immunoglobulin G1 (IgG1) (Cat# ab81032, Abcam, USA) was used as the negative control.

### Cell lines

A total of four EOC cell lines (SKOV3-Luc, 3AO, ES2 and A2780) were used for OC183B2 antigen detection and other cell-based experiments. The 3AO, ES2 and A2780 cell lines were preserved in the Center of Gynecologic Oncology, Peking University People’s Hospital. The SKOV3-Luc cell line expressing the firefly luciferase gene was kindly provided by Professor Xipeng Wang from Shanghai Jiao Tong University. SKOV3-Luc, 3AO, ES2 and A2780 cells were cultured in RPMI 1640 medium supplemented with 10% FBS and 1% penicillin and streptomycin.

### IHC staining of EOC cell lines

Four vitro cultured cell lines (SKOV3-Luc, 3AO, ES2, A2780) representing different histological types of EOC were fixed using 4% formalin after harvest. The fixed cells were then mixed with melted agarose to create a block of dispersed single cells. The resulting cell gels were subjected to standard histoprocessing and paraffin embedding. IHC staining of paraffin sections of EOC cell lines (SKOV3-Luc, 3AO, ES2, A2780) was conducted in the same way as that of EOC tissues as previously described.

### Western blot detection of SKOV3-Luc tumour samples

SKOV3-Luc tumours were excised from sacrificed mice with subcutaneously transplanted (the construction of a subcutaneous mouse tumour model of SKOV3-Luc cells is described below). Protein was extracted using RIPA buffer (Cell Signaling Technology, USA) on ice. Protein (40 μg) was subjected to 10% sodium dodecyl sulfate-polyacrylamide gel electrophoresis (SDS-PAGE). Then, protein was transferred to a 0.45 μm polyvinylidene fluoride membrane and incubated in blocking buffer (5% fat-free milk powder dissolved in phosphate-buffered saline [PBS]) for 1 h at room temperature. Membranes were incubated with primary antibodies against OC183B2 (0.2 μg/mL) and IgG1 isotype control (0.2 μg/mL, Abcam, USA) overnight at 4 °C. Membranes were imaged using an Odyssey CLx Imaging System (LI-COR Biotechnology) after incubation with IRDye800CW goat anti-mouse secondary antibody (diluted 1:10000, LI-COR Biotechnology, USA) for 1 h at room temperature.

### Development of a tumour-specific fluorescent probe

Antibodies against OC183B2 and IgG1 (Cat# ab81032, Abcam, USA) were dissolved in PBS. The concentration of antibodies was 1 mg/mL. The pH of the antibodies was raised to 8.5 by adding 1 M sodium bicarbonate buffer. IRDye800CW (Cat# 929-70021, LI-COR, USA) and antibodies were mixed (mole ratio = 1:20) and incubated overnight at 4 °C in the dark. The reaction product was purified using a Pierce Zeba desalting spin column (Cat# 87766, Thermo Fisher Scientific, USA). The dye-protein ratio was measured using a UV-Vis system after purification of the IRDye800CW-conjugated COC183B2 antibody (COC183B2-800) and IgG1 (IgG-800).

### Subcutaneous mouse model of EOC and NIRF imaging

The Medical Ethics Committee of Peking University People’s Hospital approved the study (no. 2016PHC078). Five-week-old female BALB/c nude mice were injected with 5 × 10^6^ SKOV3-luc cells in the right shoulder. Mice bearing 0.4–0.7 cm SKOV3-Luc tumours were injected with imaging agents. A total of 24 mice were used and randomly divided into 8 groups with 3 mice per group. Each group was injected with one of the following agents: (1) 50 μg COC183B2-800, (2) 25 μg COC183B2-800, (3) 12.5 μg COC183B2-800, (4) 50 μg IgG-800, (5) 25 μg IgG-800, (6) 12.5 μg IgG-800, (7) IRDye800CW alone, or (8) PBS. All agents were dissolved in 100 μl PBS and administered to mice via tail-vein injection.

To avoid fluorescent signal interference, NIRF imaging was conducted before bioluminescent imaging (BLI). From 6 to 96 h postinjection, all mice were fluorescently imaged at 800 nm to detect IRDye800CW using an IVIS Spectrum Imaging System. BLI was performed to localize SKOV3-Luc tumours. Briefly, 150 mg/kg d-luciferin was injected intraperitoneally 10 min before BLI. The tumour background ratio (TBR) was analysed by measuring the fluorescent signal of the tumour and that of normal tissue that exists around the tumour.

### Ex vivo imaging, biodistribution and histological verification

All animals were sacrificed 96 h after injection of the agents. NIRF imaging of the excised organs was performed using an IVIS Spectrum Imaging System for biodistribution studies. Histology paraffin-embedded SKOV3-Luc tumour tissues were sectioned and stained with haematoxylin-eosin (H&E). To confirm the presence of OC183B2, other sections were subjected to IHC staining using a COC183B2 antibody.

### Statistical analysis

All fluorescent signal values and TBRs of different groups and time points are depicted as the mean ± SD. Means of fluorescent signal values and TBRs were compared using one-way ANOVA or independent samples *t* test. *P* values lower than 0.05 were considered to indicate a significant difference. GraphPad Prism 5.0 (version 5.01, IBM Corp.) was used to generate graphs. SPSS 22.0 (IBM technologies) was used to perform statistical analysis.

## Results

### OC183B2 expression in EOC cell lines and human tissues

**Fig. 1:**
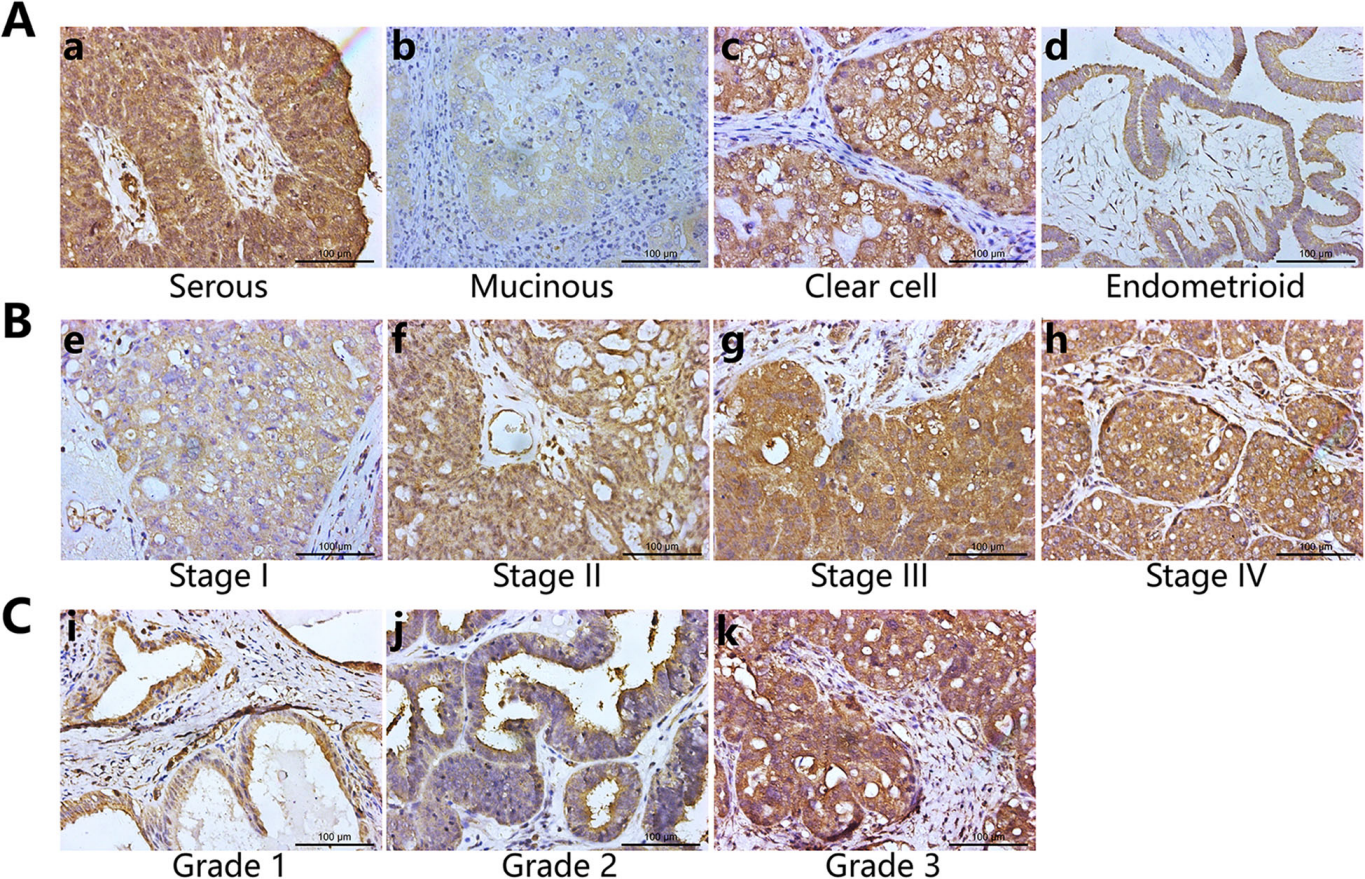
Immunohistochemical (IHC) staining of ovarian cancer tissues with COC183B2 antibody. **A** Ovarian cancer tissues of different histologic types. a, Serous ovarian cancer. b, Mucinous ovarian cancer. c, Clear cell ovarian cancer. d, Endometrioid ovarian cancer. **B** Ovarian cancer tissues of different clinical stages. e, Stage I. f, Stage II. g, Stage III. h, Stage IV. **C** Ovarian cancer tissues of different pathological grades. i, Grade 1. j, Grade 2. k, grade 3

IHC staining was conducted in the EOC tissues of 69 patients. The total positive rate of OC183B2 expression was 89.9% (62/69). We also analysed the positive rate of OC183B2 expression in samples of different histologic types, different clinical stages and different pathological grades. The expression of OC183B2 was positive in 92.2% (47/51) of serous EOCs, 25% (1/4) of mucinous EOCs, 100% (8/8) of clear cell EOCs and 100% (6/6) of endometrioid EOCs (Fig. 1A). There were 83.3% (15/18) of stage I, 100% (6/6) of stage II, 92.9% (39/42) of stage III and 66.7% (2/3) of stage IV EOC patients expressing OC183B2 (Fig. 1B). The positive rates of OC183B2 in different pathological grades were 50% (3/6) in grade 1, 100% (5/5) in grade 2 and 93.1% (54/58) in grade 3 (Fig. 1C).

**Fig. 2:**
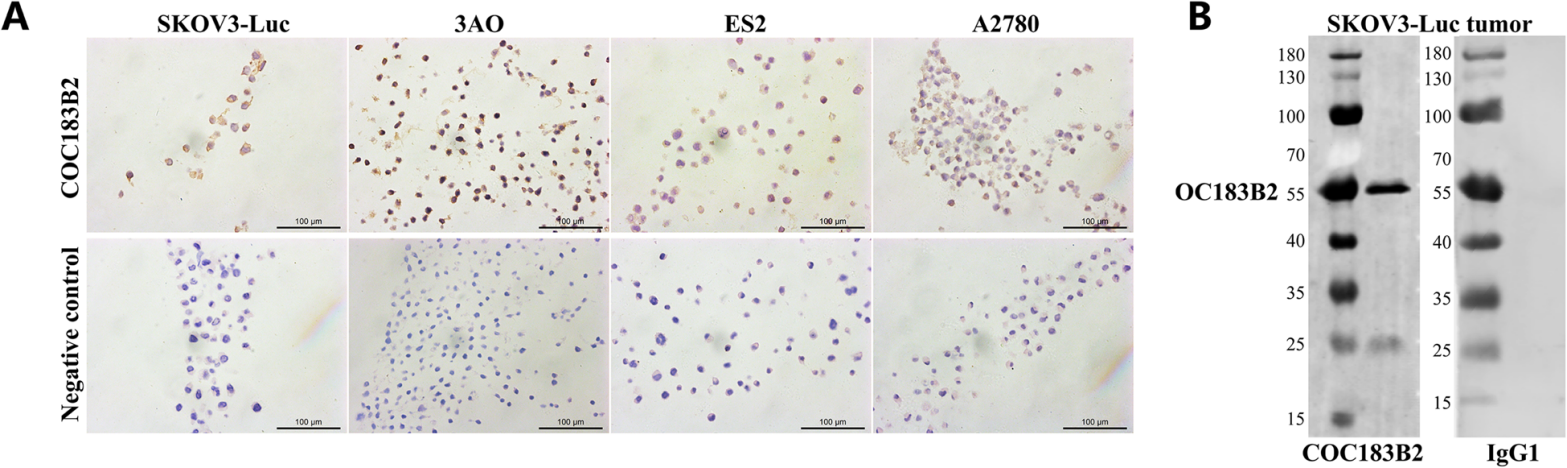
The expression of OC183B2 in ovarian cancer cell lines and SKOV3-Luc tumour samples. **a** Immunohistochemical (IHC) staining of ovarian cancer cell lines (SKOV3-Luc, 3AO, ES2, A2780). **b** Western blot analysis of OC183B2 in SKOV3-Luc tumour samples

The result of IHC staining of cell lines representing different cell types showed that OC183B2 antigen expression was positive in SKOV3-Luc (serous EOC cell), 3AO (mucinous EOC cell), ES2 (clear cell EOC cell) and A2780 (endometrioid EOC cell) cells, which is consistent with the results of IHC staining of human EOC tissues (Fig. 2a).

### Western blot detection of SKOV3-Luc tumour samples

Western blot experiments indicated that OC183B2 was expressed in SKOV3-Luc tumours. The isotype control IgG1 was negative in SKOV3-Luc tumours. The molecular weight of the OC183B2 antigen in SKOV3-Luc tumours was 56 kDa and 25 kDa (Fig. 2b), which is consistent with the results of our previous work [5].

### Development of a tumour-specific fluorescent probe

**Fig. 3:**
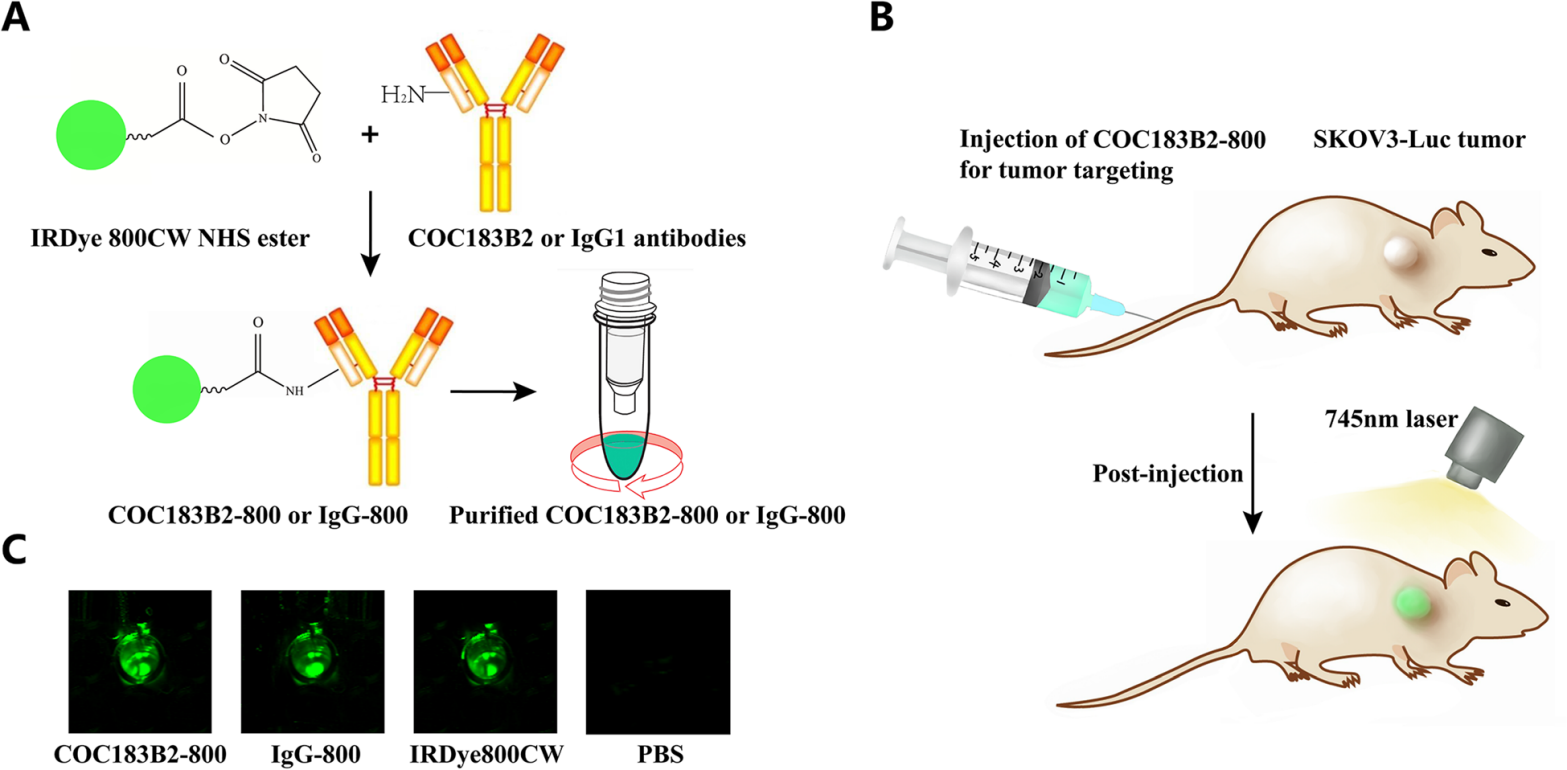
The procedure for tumour-specific NIRF imaging. **a** Conjugation and purification processes of IRDye800CW and antibodies against OC183B2 and mouse immunoglobulin G1 (IgG1) (isotype control). **b** Design of tumour-specific NIRF imaging of COC183B2-800 in a subcutaneous mouse tumour model. **c** Imaging of purified antibodies conjugated with IRDye800CW

After purification of the reaction product, antibodies conjugated with IRDye800CW were added into 96-well plates and imaged using an Odyssey CLx Imaging System. The results showed that the fluorescent signal could be detected with COC183B2-800, IgG-800 and IRDye800CW (Fig. 3c). The antibodies were conjugated with IRDye800CW successfully. The UV-Vis system confirmed that the dye-to-protein ratio of antibodies was 2.2:1.

### Specificity of COC183B2-800 in NIRF imaging in a subcutaneous mouse tumour model

**Fig. 4:**
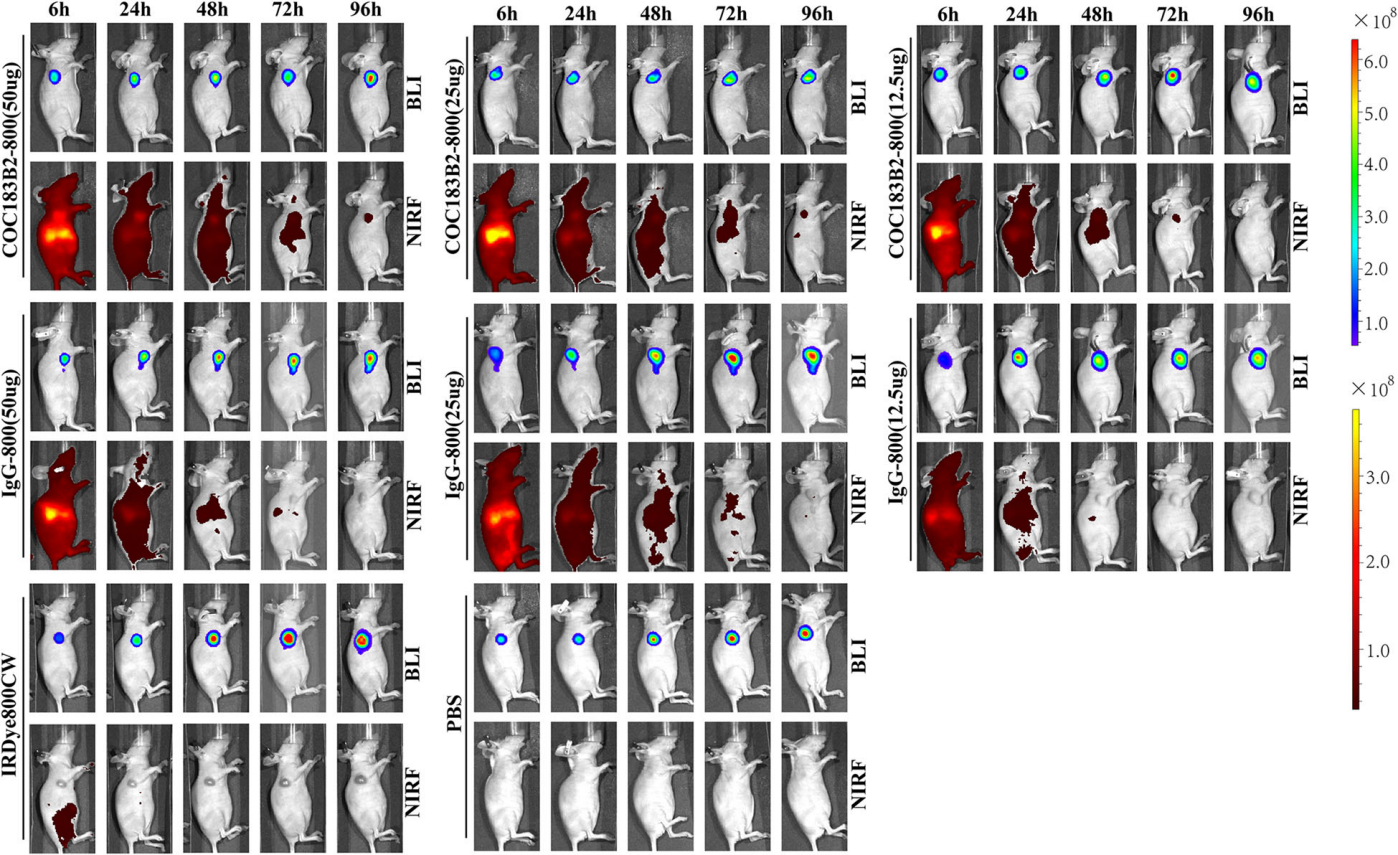
NIRF imaging and bioluminescent imaging (BLI) of different mouse groups at 6, 24, 48, 72 and 96 h postinjection

**Fig. 5:**
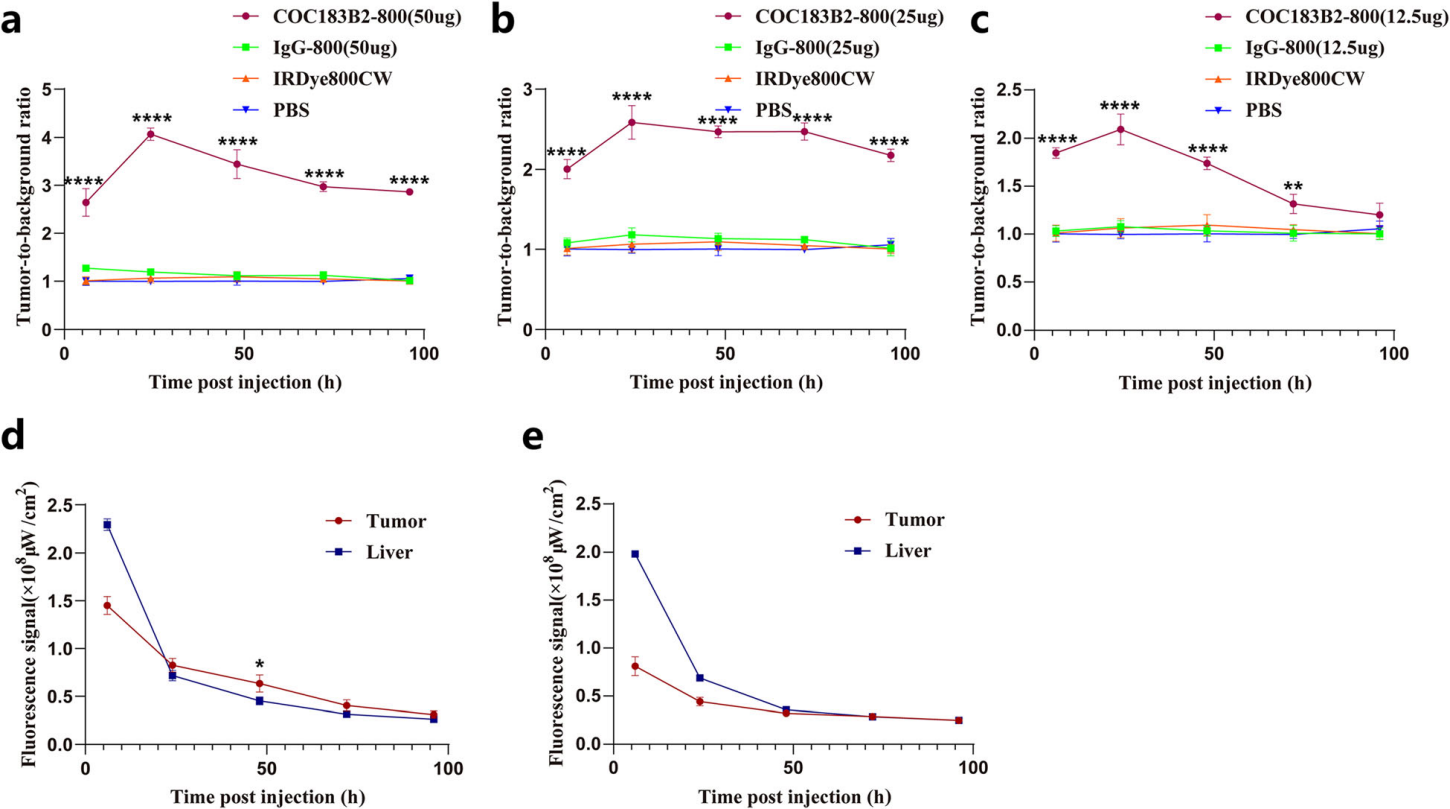
Fluorescent signal analysis of NIRF imaging of the SKOV3-Luc subcutaneous mouse tumour model. **a** TBR of 50 μg COC183B2-800, 50 μg IgG-800, IRDye800CW alone or PBS. **b** TBR of 25 μg COC183B2-800, 25 μg IgG-800, IRDye800CW alone or PBS. **c** TBR of 12.5 μg COC183B2-800, 12.5 μg IgG-800, IRDye800CW alone or PBS. **d** Fluorescent signal of the tumour and liver in the group of 50 μg COC183B2-800. **e** Fluorescent signal of the tumour and liver in the group of 50 μg IgG-800. *P < 0.05, **P < 0.01, ****P < 0.0001. Graphs are the mean ± SD

BLI was performed in all the mouse groups. NIRF imaging of the COC183B2-800 probe showed a high fluorescent signal in the tumour location, which is in line with the site found by BLI (Fig. 4). The IgG-800, IRDye800CW and PBS groups did not show tumour-specific signals (Fig. 4). The group of mice injected with 50 μg COC183B2-800 agent had a significantly (*P* < 0.0001) higher TBR than the groups injected with 50 μg IgG-800, IRDye800CW alone or PBS at 6 h (*P* < 0.0001), 24 h (*P* < 0.0001), 48 h (*P* < 0.0001), 72 h (*P* < 0.0001) and 96 h (*P* < 0.0001) postinjection (Fig. 5a). The TBR of mice injected with 25 μg COC183B2-800 was significantly higher than that of mice injected with 25 μg IgG-800, IRDye800CW alone or PBS at 6 h (*P* < 0.0001), 24 h (*P* < 0.0001), 48 h (*P* < 0.0001), 72 h (*P* < 0.0001) and 96 h (*P* < 0.0001) after injection (Fig. 5b). There was a significantly higher TBR in the 12.5 μg COC183B2-800 group than in the 12.5 μg IgG-800, IRDye800CW alone or PBS groups at 6 h (*P* < 0.0001), 24 h (*P* < 0.0001), 48 h (*P* < 0.0001) and 72 h (*P* = 0.002) after injection (Fig. 5c). There was increase in TBR as the dose of COC183B2-800 increased. High fluorescent signals were mainly concentrated in the liver, and we compared the fluorescent signals of the liver and tumour in the COC183B2-800 and IgG-800 groups. The fluorescent signal of the tumour was significantly higher than that of the liver in the 50 μg COC183B2-800 group at 48 h postinjection (*P* = 0.0361; Fig. 5d).

### Ex vivo imaging, biodistribution and histological verification

**Fig. 6:**
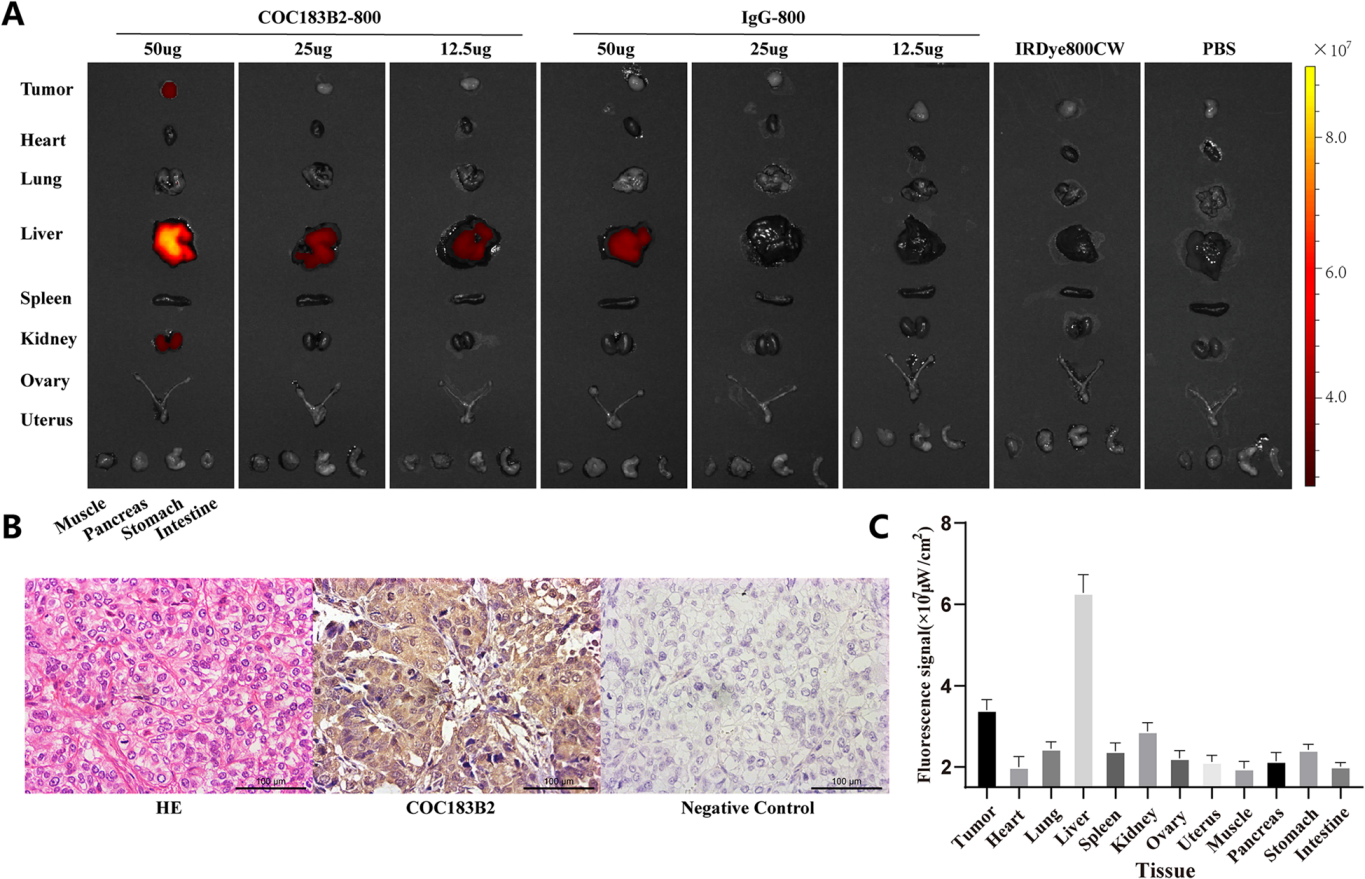
NIRF imaging of organs and histological verification of SKOV3-Luc tumours. **a** Fluorescent imaging of tumours and different organs of each group at 96 h postinjection. **b** Haematoxylin-eosin (H&E) staining of SKOV3-Luc tumours and immunohistochemistry (IHC) staining of tumours with COC183B2 antibody. **c** Fluorescent signals of tumours and different organs in the 50 μg COC183B2-800 group

After the mice were sacrificed, the organs were excised. The results of H&E staining verified the excised tumour tissue. IHC staining of OC183B2 was positive in SKOV3-Luc tumours (Fig. 6b). Ex vivo NIRF imaging showed that there was still a strong fluorescent signal in the tumours of the 50 μg COC183B2-800 group (Fig. 6a). In the other groups, a strong fluorescent signal could only be detected in the liver (Fig. 6a). In the PBS control group, the lowest fluorescent signal that could be detected is 1.5 × 10^7^ μW/cm^2^. Biodistribution assays of the 50 μg COC183B2-800 group showed that the highest fluorescent signal was in the liver, and the second-highest fluorescent signal was in the tumour (Fig. 6c). The fluorescent signal was slightly higher in the kidney than in the rest of the organs. Relatively low fluorescent signals could be detected in other organs (heart, lung, spleen, ovary, uterus, muscle, pancreas, stomach and intestine) (Fig. 6c). In the IgG-800 group, the fluorescent signal in the liver was low, which may be caused by the shorter fluorescence lifetime of the nonspecific probe of IgG-800 compared with that of the specific antibody probe [11].

## Discussion

To improve the survival of EOC patients, it is crucial to reduce the presence residual lesions after surgery. Currently, surgeons mainly depend on visual inspection and palpation to identify lesions. Conventional imaging modalities, such as MRI, CT and PET/CT, have a limitation of low sensitivity and specificity. Subcentimetre lesions are usually difficult to identify by CT and MRI. PET/CT is usually performed to determine the site of metastasis before surgery for recurrent cancer. However, PET/CT is not sensitive enough to detect clear cell or mucinous carcinoma, tumours with postchemotherapy metabolic inhibition, cystic lesions and tiny lesions, which are factors that affect surgical decisions. Our immunohistochemical experiments showed that clear cell carcinomas expressed OC183B2. Theoretically, COC183B2-800 probes can identify such tumours. Moreover, conventional imaging modalities cannot be performed intraoperatively or guide surgery by imaging. Surgeons may miss subcentimetre lesions and metastatic lesions [12]. To overcome the limitations of conventional imaging modalities and guide surgery, highly specific intraoperative in vivo fluorescent imaging that has the advantages of high specificity, superior resolution and real-time detection is needed. Tumour-specific fluorescence-guided surgery could guide the resection of tumour-positive margins, tiny lesions and tumour-positive lymph nodes and improve survival by reducing the presence of residual disease. Previously, Hutteman et al. used indocyanine green (ICG), a nontargeted optical imaging agent, to detect pancreatic ductal adenocarcinoma. However, after intravenous injection of ICG, no useful tumour demarcation could be visualized [13]. ICG lacks imaging specificity, which will lead to the misdiagnosis of positive lesions. Therefore, targeted optical imaging, which depends on the binding of antibodies to antigens or ligands to receptors, is needed to replace nontargeted optical imaging. Clinical trials of targeted tumour-specific fluorescence imaging conducted in different malignancies have shown remarkable success [14–17]. Though ICG can be used in humans, it lacks active groups to conjugate with ligands or antibodies. Most tumour-specific fluorescent probes use IRDye800CW since IRDye800CW has already been extensively tested in clinical studies and can be conjugated to ligands or antibodies [18].

In previous studies, the tumour targets of ovarian cancer-specific fluorescence imaging included folate receptor alpha (FR-α) [19, 20], follicle-stimulating hormone receptor (FSHR) [2], anti–human epidermal growth factor receptor 2 (HER2) [21] and so on. However, the widespread expression of HER2 in normal human tissues impacts the specificity and accuracy of imaging [22]. The relatively low expression rate (56.4%) of FSHR may impact the universality of imaging [23]. In a clinical trial of fluorescence imaging based on the target FR-α, false-positive fluorescence was identified in 17/50 nonmetastatic lymph nodes caused by OTL-38 targeting of FRβ, which is expressed by tumour-associated activated macrophages [24]. Therefore, to better improve ovarian cancer-specific fluorescence-guided surgery, it is necessary to seek and develop better tumour targets or targets that could be functionally complementary to known targets. We previously developed the monoclonal antibody COC183B2, which has high-affinity binding to EOC tissue. IHC staining was conducted in the human EOC tissues of 69 patients. The total positive rate of OC183B2 expression was 89.9% (62/69). The expression of OC183B2 could be detected in different histologic types, different clinical stages and different pathological grades of EOC. Therefore, the COC183B2-800 fluorescence probe has extensive applicability in a wide range of EOC patients. Furthermore, the OC183B2 antigen is rarely expressed in normal tissues, guaranteeing the specificity of imaging [6]. Our previous study of radioimmunoimaging via ^131^I-COC183B2 in patients suggests that the COC183B2 antibody can be used for radioimmunoimaging to identify the location of ovarian cancer [6].

However, both imaging probes and the equipment for imaging of ^131^I-COC183B2 have a radiation risk. Moreover, the lesions of patients are not visible during the operation due to the imaging modality. We later conjugated ultrasmall superparamagnetic iron oxide nanoparticles (USPIOs) and Cy7 with COC183B2 antibody and achieved early detection of EOC through OC183B2-targeted MRI and fluorescence imaging [10, 25]. However, antibodies conjugated with USPIOs might be toxic. Cy7 has not been approved for use in humans, and our previous work showed the phenomenon of lung accumulation of Cy7-conjugated COC183B2 [10]. As previously described, IRDye800CW is a safe and effective NIRF dye. Therefore, we conjugated COC183B2 to IRDye800CW and synthesized COC183B2-800. The results of IHC staining of cell lines representing different cell types showed that the OC183B2 antigen expression was positive in SKOV3-Luc (serous EOC cell), 3AO (mucinous EOC cell), ES2 (clear cell EOC cell) and A2780 (endometrioid EOC cell) cells, which is consistent with the result of IHC staining of human EOC tissues. SKOV3-Luc cells represent serous EOC which accounts for approximately 70% of all EOCs. IHC staining intensity of different cell types showed that the expression of OC183B2 in SKOV3-Luc cells was relatively higher, and the western blot results also showed that OC183B2 was expressed in SKOV3-Luc tumours. Thus, the SKOV3-Luc cell line was suitable for further study in a subcutaneous mouse model.

The NIRF imaging of the COC183B2-800 probe showed a high fluorescent signal at the tumour location, which was in line with the site detected by BLI. COC183B2-800 showed excellent tumour-specific targeting in the subcutaneous mouse tumour model. At different time points (6, 24, 48, 72 and 96 h postinjection), mice in the groups with 50 μg and 25 μg COC183B2-800 all showed significantly higher TBR than the corresponding groups of IgG-800, IRDye800CW and PBS. In the 12.5 μg COC183B2-800 group, TBR was significantly higher at 6, 24, 48 and 72 h than it was in the other groups, but this effect was not observed at 96 h. The TBR declined as the dose of COC183B2-800 was reduced. The proper dose of COC183B2-800 for imaging may be 25~50 μg. According to the change in TBR over time, 24~72 h postinjection may be the best period of time for imaging. Furthermore, the fluorescent probe COC183B2-800 was metabolized mainly through the liver and did not accumulate in other organs. COC183B2-800 is a very safe and effective ovarian cancer-specific fluorescent probe.

## Conclusions

In conclusion, we developed the novel ovarian cancer-specific antibody fluorescent probe COC183B2-800. Through the evaluation of COC183B2-800 in a subcutaneous mouse model of EOC, we found that COC183B2-800 could bind selectively to the EOC antigen OC183B2. COC183B2-800 exhibited excellent TBR, and there was a clear correlation between the histopathologic evidence and the fluorescent signal. Ovarian cancer-specific imaging was achieved, with favourable liver and kidney clearance. IRDye800CW has already been proven safe to be used in humans, and our experiment paves the way for clinical translation of the fluorescence imaging agent COC183B2-800.

## Data Availability

Not applicable.

## Abbreviations

EOC: Epithelial ovarian cancer
IHC: Immunohistochemistry
IgG1: Immunoglobulin G1
NIRF: Near-infrared fluorescence
BLI: Bioluminescent imaging
TBR: Tumour background ratio
MRI: Magnetic resonance imaging
CT: Computed tomography
